# Sales of oseltamivir in Norway prior to the emergence of oseltamivir resistant influenza A(H1N1) viruses in 2007–08

**DOI:** 10.1186/1743-422X-6-54

**Published:** 2009-05-12

**Authors:** Siri H Hauge, Hege S Blix, Katrine Borgen, Olav Hungnes, Susanne G Dudman, Preben Aavitsland

**Affiliations:** 1 Department of Infectious Disease Epidemiology, Norwegian Institute of Public Health, PO Box 4404 Nydalen, N-0403 Oslo, Norway; 2 Norwegian Field Epidemiology Training Programme (NorFETP), Oslo, Norway; 3 Department of Pharmacoepidemiology, Norwegian Institute of Public Health, PO Box 4404 Nydalen, N-0403 Oslo, Norway; 4 Department of Virology, Norwegian Institute of Public Health, PO Box 4404 Nydalen, N-0403 Oslo, Norway

## Abstract

**Background:**

An unprecedented high proportion of oseltamivir resistant influenza A(H1N1) viruses emerged in the 2007–08 influenza season. In Norway, two thirds of all tested A(H1N1) viruses were resistant to the antiviral drug. In order to see if this emergence could be explained by a drug induced selection pressure, we analysed data on the sales of oseltamivir in Norway for the years 2002–07.

**Methods:**

We used data from two sources; the Norwegian Drug Wholesales Statistics Database and the Norwegian Prescription Database (NorPD), for the years 2002–2007. We calculated courses sold of oseltamivir (Tamiflu^®^) per 1000 inhabitants per year.

**Results:**

Our data showed that, except for the years 2005 and 2006, sales of oseltamivir were low in Norway; courses sold per 1000 inhabitants varied between 0.17–1.64. The higher sales in 2005 and 2006 we believe were caused by private stockpiling in fear of a pandemic, and do not represent actual usage.

**Conclusion:**

A drug induced selection pressure was probably not the cause of the emergence of oseltamivir resistant influenza A(H1N1) viruses in 2007–08 in Norway.

## Background

The 2007–08 influenza season on the Northern Hemisphere was characterized by an unprecedented high proportion of influenza A(H1N1) viruses resistant to the antiviral drug oseltamivir[1]; a neuraminidase inhibitor used as prophylaxis or treatment for influenza. This development was first detected in and reported by Norway. By the end of the 2007–08 influenza season in Norway[2], two thirds of all A(H1N1) viruses tested were resistant against oseltamivir, the highest proportion recorded in any country on the Northern Hemisphere[3].

The oseltamivir resistance was caused by a known mutation causing a histidine to tyrosine substitution at the position 275 in the viral N1 neuraminidase gene. This substitution is associated with a high-level resistance to oseltamivir[4]. The mutation had previously been found in less than 1% of influenza A viruses tested[5] and had been associated with low viral fitness and reduced ability to transmit[6].

Cross-resistance towards another neuraminidase inhibitor zanamivir has previously been shown, but not with this particular mutation. In accordance with this, the A(H1N1) viruses detected during the 2007–08 season remained susceptible to zanamivir.

Many countries, including Norway, have stockpiled oseltamivir as a part of the pandemic preparedness, according to WHO recommendations[7]. In Norway, oseltamivir has been available as a prescription-only drug since June 2002[8], and is licensed for persons older than one year. One course equals a five-day treatment with 75 mg × 2 daily. The price of one course is approximately 24 € or 34 USD (January 2009). In order to see if the emergence of the high proportion of oseltamivir resistant influenza viruses in Norway in 2007 was caused by a drug induced selection pressure, we analysed data on the sales of oseltamivir in Norway for the years 2002–07.

## Methods

We used two different sources of information on sale figures of oseltamivir (Tamiflu^®^).

Firstly, we extracted data from the Norwegian Drug Wholesales Statistics Database. This database is administered by the Norwegian Institute of Public Health and contains complete data on all medicines sold from the wholesalers to Norwegian pharmacies, hospitals and nursing homes. Information of sales is available as packages sold and as number of defined daily doses . We used population data from Statistics Norway  to calculate courses sold per 1000 inhabitants. We also extracted data about sold courses of zanamivir (Relenza^®^) from this database.

Secondly, we used data from the Norwegian Prescription Database (NorPD). This database was established in 2004 and is administered by the Norwegian Institute of Public Health. All Norwegian pharmacies report all prescriptions filled by outpatients. Thus, these numbers represent a subset of the data in the Wholesales Statistics. The prescriptions can be traced to individuals using a unique personal identification number. However, a small minority of prescriptions lacks this number. In this study we included all prescriptions, with or without an id number, with the assumption that it is unlikely that a person would obtain oseltamivir several times during one year.

## Results

We found that oseltamivir sales in Norway in the years 2004–7 varied between 0.17 – 1.64 courses per 1000 inhabitants per year, except for the years 2005 and 2006 (table 1).

**Table 1:** Courses sold of oseltamivir in Norway 2004–2007, data from the Norwegian Drug Wholesales Statistics Database and the NorPD

	**Wholesale Statistics**		**NorPD**	
	
**Year**	**Number of courses sold**	**Courses sold pr. 1000 inhabitants**	**Number of courses sold**	**Courses sold pr. 1000 inhabitants**
2002*	864	0.19	Data not available	Data not available
**2003**	7465	1.64	Data not available	Data not available
**2004**	766	0.17	764	0.17
**2005**	65258	14.17	23328	5.06
**2006**	33006	7.11	4839	1.04
**2007**	4561	0.97	3478	0.74

*Oseltamivir in sale from June 2002.

In the same period, zanamivir was sold in very low numbers according to the Wholesales Statistics: 2004: 54 courses, 2005: 51 courses, 2006: no courses sold, 2007: 7 courses.

**Figure 1:**
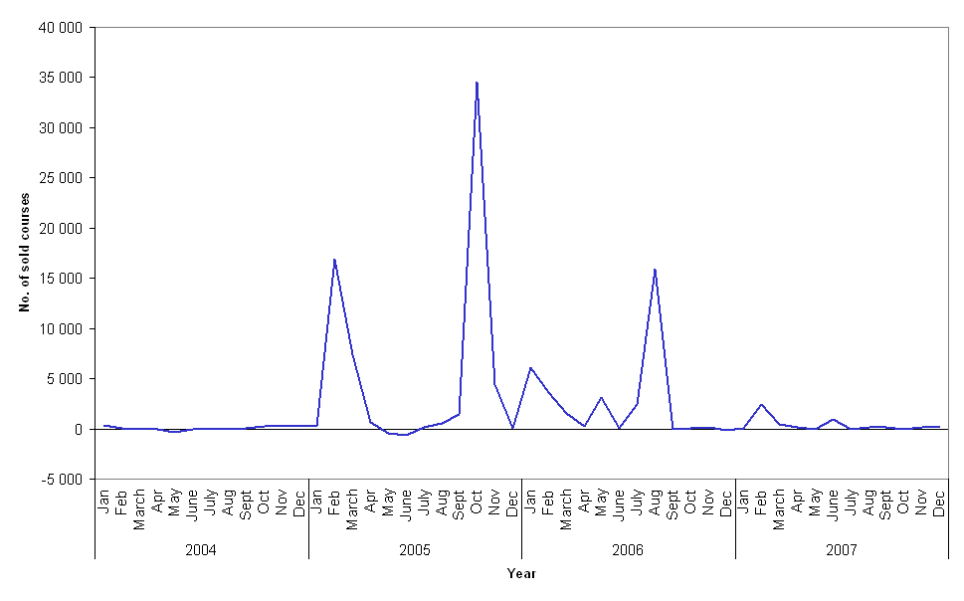
**Courses sold of oseltamivir in Norway for the years 2004–2007**. Numbers below zero indicate return of the drug from pharmacies to the wholesaler. Source: Norwegian Drug Wholesales Statistics.

Data from the Wholesales Statistics showed that the high sales in 2005 and 2006 mainly were due to high sales in February and October 2005, and in August 2006 (figure 1).

## Discussion

Our results show that oseltamivir sales in Norway were low prior to the 2007 emergence and widespread circulation of oseltamivir resistant influenza A(H1N1) viruses. Thus, the emergence of oseltamivir resistance does not seem to be caused by a drug induced selection pressure in Norway. Furthermore, the persistence of resistance throughout the season indicates that the resistant viruses sustained their fitness independently from a selection pressure by oseltamivir.

We have no method for measuring the actual usage of oseltamivir, but we believe that the higher sales and prescription figures in 2005 and 2006 can be explained by the public's stockpiling in fear of a pandemic, and does not represent actual usage in this period. This is supported by the lack of relationship between increased influenza activity[9] and the highest peaks of sales of oseltamivir in 2005 and 2006. In 2005 there was an increased media attention on pandemic flu, and private stockpiling of oseltamivir was causing empty pharmacies in the beginning of the year. In the US, increased media attention on the pandemic flu also caused private stockpiling of the drug outside the influenza-season[10]. In November 2005 the Norwegian authorities issued an official advice against private stockpiling of oseltamivir[11].

The difference in numbers from the Wholesales Statistics and NorPD in 2005–06 might be explained by deviation from normal dispensing rules by many pharmacies, because of the mass of total demand in this period. The consequence was that the complete sales of oseltamivir were registered in the Wholesales Statistics and not in the NorPD.

Privately imported drugs following Internet purchases are not included in our figures, but we believe this represent a very small amount. Similarly, there may have been some, but probably not widespread, usage of privately stockpiled drug during subsequent influenza seasons, with or without medical consultation.

## Conclusion

Our assumption is that use of oseltamivir in Norway was low prior to the emergence of oseltamivir resistant influenza viruses, as shown by the low sales figures except for 2005 and 2006 when private stockpiling most likely caused the higher sales. The emergence and widespread circulation of the oseltamivir resistant influenza A(H1N1) virus in the 2007–08 season was probably not caused by a drug induced selection pressure in Norway.

## Competing interests

The authors declare that they have no competing interests.

## Authors' contributions

SHH participated in the initiation of the data collection, analysis and drafting of the manuscript; HSB collected and analysed data from the Wholesales Register and NorPD and participated in drafting of the manuscript; KB, OH and SD all participated in the drafting of the manuscript and PA participated in the initiation of the data collection, analysis and drafting of the manuscript.
